# Author’s Response to Peer Reviews of “Effect of Thermal and Vibration Changes on Automated External Defibrillator Circuit Boards: Finite Element Analysis Study”

**DOI:** 10.2196/80135

**Published:** 2025-08-19

**Authors:** Saidi Olayinka Olalere

**Affiliations:** 1 Department of Mechanical Engineering, Georgia Southern University, 1332 Southern Drive, Statesboro, GA, 30458, United States, 1 9124784636

**Keywords:** circuit board, automated external defibrillator, heart, cardiology, vibration, thermal changes, medical devices

*This is the authors’ response to peer-review reports for “Effect of Thermal and Vibration Changes on Automated External Defibrillator Circuit Boards: Finite Element Analysis Study.”*

## Round 1 Review

### Reviewer R [1]

#### General Comments

*This paper [2] considered the vibration and thermal analysis of a modeled circuit board of an automated external defibrillator (AED) using Ansys. The vibration failure in the modeled circuit board with four rigid supports was pronounced starting at the taller components, including the capacitor. The board was reinforced to an 8–rigid support system, reducing the failure around the rigid supports. The thermal failure started from the battery position, causing thermal dissipation to other parts of the board, ultimately leading to the failure of the circuit board and the AED.*

#### Specific Comments

##### Major Issues

*1. “The goal is to analyse the effect of vibration and thermal experience on the AED based on its operation.” Is this your research statement? I suppose the topic suggests you analyzed the modeled circuit board, not the overall AED system. If not, how did you measure the overall effect on the AED?*

**Response:** The research was a simulation of how a modeled circuit board of AED is affected by vibration and thermal conditions. This was the basis of the research, and the analysis was performed on these premises.

*2. The author may also need to discuss the importance of the circuit boards in an AED in the Introduction.*

**Response:** The AED circuit board for the analysis will be a material made of epoxy FR-4 with a length of 254 mm and width of 216 mm, while the thickness is 0.5 mm. The components of the circuit board include a capacitor, microcontroller, flash memory, analogue digital converter, field programmable gate array, processor, audio controller, inductor, and more.

*3. The figures will need a little more discussion.*

**Response:** The figures were explained individually. Also, a Discussion heading was created to discuss the findings before a conclusion was used to end the paper.

##### Minor Comments

*4. “Vibration and Thermal Analysis on Modeled Circuit Board of Automated External Defibrillator (AED) Medical Device” will likely communicate the title better.*

*5. I find it a bit difficult to understand this line: “Fatigue failure under sinusoidal vibration loading for component by comparing the vibration failure test, FEA, and theoretical test (Y.S.Chen, 2008).” What did you want to say?*

**Response:** This is a research reference to Chen et al [3], who explained that failure was experienced from vibration loading on components when the simulation and experimental testing were performed.

*6. Figure 1 will need relabeling. The labeling seems to cover some parts of the board. A transparent background could help.*

**Response:** The majority of the labels are outside of the board, and a transparent background could distort the labeling and look like wording on the board.

### Anonymous [4]

#### General Comments

*I have read the submitted manuscript to your journal entitled “Analysis of Vibration and Thermal of a Modeled Circuit Board of Automated External Defibrillator (AED) Medical Device.” The author utilized finite element analysis to simulate static and dynamic testings in determining the vibration and thermal effects on the operations of the AED medical device.*

*Based on the outcomes of this conducted study and the potential benefits from this study, I would recommend this manuscript to be published in your reputable journal after the minor comments are properly addressed.*

#### Specific Comments

##### Minor Comments

*There are 4-member and 8-member support. The author should provide more evidence on how these affect the analysis results.*

**Response:** A physical AED was used as a prototype to model the circuit board. A physical AED is always a 4-member support, which was the basis of the research. For further understanding, an 8-member support was modeled to understand any change with respect to the vibration with a change in the support.

*This manuscript will benefit from other works on the failure modes of materials. The author is encouraged to expand the Literature Review section and also provide more references.*

**Response:** The literature has been expanded and necessary references included.

*The grammar should be refined. Some of the grammar in this manuscript should be corrected. For example, “This study was performed to analysis the vibration...” “Analyse.” Fig 14 = Fig. 14 or Figure 14. The unit of temperature is degree c; the c is capitalized.*

**Response:** The grammatical errors in the abstract and figures have been corrected.

*When citing a research paper within the manuscript, it is the first/lead author who should be named in the “Name et al” format; the author should use a consistent citation format.*

**Response:** The citation has been corrected.

*The author should remove parentheses from the manuscript title: “Analysis of Vibration and Thermal of a Modeled Circuit Board of Automated External Defibrillator Medical Device.”*

**Response:** Parentheses have been removed from the title.

*For reproducibility by other researchers, the author should consider providing simulation data as supplementary information.*

**Response:** For reproducibility, the materials and methods provide the necessary information methodology and requirements in terms of model design, mesh selection, variables and values, and more.

*The author should properly cite other works where applicable. For example, “From other research results, it can be verified that the natural frequencies...” The author needs to insert appropriate citations for comments like these.*

**Response:** The necessary citation has been included as recommended.

## Round 2 Review

### Reviewer R

*1. The recommendation about explaining how the author measured the overall effect of the analysis on the AED or the board and the recommendation that the author should provide more evidence on how the 4-member and 8-member supports affect the analysis result have not been answered or addressed in the manuscript. The author should consider these.*

**Response:** The 4 and 8 members were explained more with tables and figures, which can be found in the manuscript. The 8 members were explicated in Figures 12 and 13.

*2. The author will also need to be consistent. Is Figure 5 the same as Figure 5 or Fig. 5? It should be corrected for all other instances.*

**Response:** This is a generally acceptable concept in manuscripts, where Figure (in full) is used in the naming convention, while Fig. is used in further explanation.

*3. Additional citations might be needed in the work; it still looks like over 40% of the citations are 15 years or older. Also, “et al.,” should be in italics with a period after the “al.” The Discussion also seems not well discussed in relation to previous works.*

*4. The template format should also be considered carefully.*

**Response:** Only related citations were included in the manuscript. This manuscript shows a blend of early research and recent research that aligned with the purpose of the paper. I have 10 references that are more detailed toward the research.

## Abbreviations

AED: automated external defibrillator
